# 

**Published:** 1859-02

**Authors:** 


### CONTENTS.

ORIGINAL.

Physiological Observations on the Teeth, by Prof. St. John........................ 141
Treatment of Dental Pulp................ 147
Singular Phenomena—Reproduction of Enamel, &c., &c.,.................... 150
Destroying the Nerve and Fang Filling, by Joseph Mansfield,................... 152
Electrical Anaesthesia,................. 154
Finishing, Fillings, etc., by A. F. Ennis, 155
Continuous Gum Work, by W. B. Roberts 161
Michigan State Dental Association,...... 180

SELECTED.

Anaesthetics,.. ...................... 191
Fractures of the Lower Jaw,............ 193
The Scientific Artizan,.................. 195
The Vulcanite Base..................... 196
Ladies in the Dental Profession,......... 196
Every man his own Dentist,............. 196
The Cincinnati Medical News,............ 197
Apology,.............................. 199
The Dental Reporter,................... 200
Dental Register,........................ 201
Galvanism in extracting Teeth,......... 202

